# Implausible 100–400 m Performance Times and Inadequate Testing Distances Compromise the Estimation and Interpretation of Critical Velocity. Comment on Amara et al. Relationships Between Dry-Land Load—Velocity Parameters and In-Water Bioenergetic Performance in Competitive Swimmers. *Sports* 2026, *14*, 11

**DOI:** 10.3390/sports14100419

**Published:** 2026-09-29

**Authors:** Ricardo J. Fernandes, Jose A. Abraldes

**Affiliations:** 1 Centre for Research, Education, Innovation and Intervention in Sport (CIFI2D) and Porto Biomechanics Laboratory, Faculty of Sport, University of Porto, 4200-450 Porto, Portugal; 2 Research Group Movement Sciences and Sport (MS & SPORT), Department of Physical Activity and Sport, Faculty of Sport Sciences, University of Murcia, 30100 Murcia, Spain

Amara et al. recently examined the relationships between upper-body bench press load–velocity profile parameters and in-water bioenergetic indicators in competitive swimmers, with particular emphasis on critical velocity and the distance–time model intercept, interpreted by the authors as anaerobic capacity [1]. The research question is relevant because the integration of dry-land neuromuscular assessment with swimming-specific physiological testing may provide useful information for performance monitoring and training prescription [2]. However, two issues in the published article require formal clarification because they directly affect the validity of the critical velocity estimates, the associated intercept values and all subsequent correlation and regression analyses derived from these variables.

The first concern relates to the plausibility of the reported front crawl performance times. The authors report mean times of 29.35 ± 1.43 s for 100 m, 66.30 ± 3.23 s for 200 m and 140.88 ± 6.90 s for 400 m, and provide an individual example for “swimmer 1” with corresponding times of 29.09 s, 65.86 s and 139.39 s [1]. These values imply average swimming velocities of approximately 3.41, 3.02 and 2.84 m·s^−1^ for the group mean performances (respectively). For comparison, the official men’s long-course world records for the 100, 200 and 400 m freestyle correspond to substantially lower average velocities, confirming that the reported values exceed current world record-level performances for all three distances [3]. This issue is particularly important because the participants were described as 16.5 ± 0.31-year-old regional- to national-level male swimmers rather than senior world-class athletes [1].

The individual example presented in the article further illustrates the problem. Using the reported times of 29.09, 65.86 and 139.39 s for 100, 200 and 400 m, the authors obtained a distance–time regression of y = 2.7199x + 20.857, corresponding to a critical velocity of 2.72 m·s^−1^ and an intercept of 20.86 m [1]. The resulting critical velocity value exceeds even the average velocity corresponding to the official men’s 100 m freestyle world record and cannot plausibly represent a sustainable predominantly aerobic swimming velocity [3]. In contrast, an intercept of 20.86 m is not intrinsically implausible when considered in isolation, as previous research using 50, 100 and 400 m trials reported anaerobic distance capacity values ranging from 18.3 to 20.5 m [4]. However, this numerical similarity does not validate the value reported by Amara et al., because the intercept is derived from the same distance–time regression and is, therefore, compromised when the underlying performance times are incorrectly reported. Moreover, anaerobic distance capacity is sensitive to the number and length of the trials used in its calculation, and its validity as a physiological measure of anaerobic capacity remains uncertain [4,5]. Consequently, the reported associations between critical velocity, anaerobic capacity and dry-land load–velocity parameters cannot be interpreted reliably until the original time data are verified and, if necessary, corrected.

A second concern relates to the methodological validity of estimating critical velocity exclusively from short-distance maximal trials. Amara et al. used 100, 200 and 400 m performances to derive critical velocity and the distance–time intercept [1]. These distances are performed in exercise intensity domains that entail a substantial anaerobic contribution [6], which should be carefully considered when derived parameters are interpreted as markers of aerobic capacity, endurance performance or training prescription [7]. Even if the reported times were corrected, the exclusive reliance on these short-distance trials remains problematic for the physiological interpretation attributed to critical velocity. Previous methodological work has also emphasised that swimming critical velocity is influenced by the choice of test distances and that the absence of a sufficiently long trial may lead to systematic overestimation and reduced physiological validity [8,9].

This distinction is not merely technical. If critical velocity is interpreted as an index of long-duration aerobic performance or as a robust marker for endurance-related training prescription, the testing protocol must be consistent with that physiological meaning. Short-distance protocols may still provide useful performance-derived parameters, but they should not support strong conclusions about aerobic capacity unless their limitations are explicitly recognised. In the current case, the use of 100, 200 and 400 m trials, without at least one sufficiently long maximal bout, weakens the interpretation of critical velocity as a robust aerobic marker and also compromises the interpretation of the intercept as anaerobic capacity. This is especially relevant because the article uses these derived variables as central outcomes in its correlation and regression analyses.

Accordingly, we suggest that the published record should be corrected or clarified, particularly by: (i) explaining the origin of the reported 100, 200 and 400 m performance times and providing corrected descriptive statistics if reporting or data-entry errors occurred; (ii) recomputing the individual distance–time model parameters, including critical velocity and the model intercept, using verified performance data; (iii) updating all correlation and regression analyses, figures and tables that depend on these variables; and (iv) explicitly acknowledging the limitations of estimating critical velocity from short-distance trials only. If no longer maximal trial, such as an 800 or 1500 m swim, is available in the dataset, the authors should avoid physiological interpretations of critical velocity that depend on long-duration validity.

In summary, the article addresses an applied and potentially useful question, but the reported 100–400 m performance times and the short-distance-only estimation protocol raise substantive concerns about the validity of its main bioenergetic variables. These issues affect not only the descriptive values of critical velocity and the model intercept, but also the central inferential claims relating dry-land neuromuscular characteristics to in-water aerobic and anaerobic indicators. Formal clarification and, if necessary, correction are welcome to preserve the accuracy and interpretability of the scientific record.

## Data Availability

No new data were created or analyzed in this study. Data sharing is not applicable to this article.

## References

[1] Amara S., Bouassida A., Tillaar R.v.d. (2026). Relationships Between Dry-Land Load–Velocity Parameters and In-Water Bioenergetic Performance in Competitive Swimmers. Sports.

[2] Carvalho D.D., Goethel M.F., Erblang M., Vilas-Boas J.P., Pyne D.B., Fernandes R.J., Lopes P. (2026). Impact of an overload period on heart rate variability, sleep quality, motivation and performance in high-level swimmers: Use of explainable artificial intelligence (XAI) to assess training load variations. Sports Med..

[3] World Aquatics. Swimming World Records. https://www.worldaquatics.com/swimming/records.

[4] Dalamitros A.A., Fernandes R.J., Toubekis A.G., Manou V., Loupos D., Kellis S. (2015). Is speed reserve related to critical speed and anaerobic distance capacity in swimming?. J. Strength Cond. Res..

[5] Scott B.E., Burden R., Dekerle J. (2023). Stroke-specific swimming critical speed testing: Balancing feasibility and scientific rigour. J. Hum. Kinet..

[6] Vescovi J.D., Falenchuk O., Wells G. (2011). Blood lactate concentration and clearance in elite swimmers during competition. Int. J. Sports Physiol. Perform..

[7] Fernandes R.J., Carvalho D.D., Figueiredo P. (2024). Training zones in competitive swimming: A biophysical approach. Front. Sports Act. Living.

[8] Fernandes R.J. (2018). Swimming Critical Velocity Physiological Meaning is Affected by Testing Distances. Int. Physiol. J..

[9] Wright B., Smith D.J., Miyashita M., Mutoh Y., Richardson A.B. (1994). A protocol for the determination of critical speed as an index of swimming endurance performance. Medicine and Science in Aquatic Sports.

